# Can public online databases serve as a source of phenotypic information for *Cannabis* genetic association studies?

**DOI:** 10.1371/journal.pone.0247607

**Published:** 2021-02-23

**Authors:** Matthew L. Aardema, Rob DeSalle

**Affiliations:** 1 Department of Biology, Montclair State University, Montclair, New Jersey, United States of America; 2 Sackler Institute for Comparative Genomics, American Museum of Natural History, New York, New York, United States of America; Texas Tech University, UNITED STATES

## Abstract

The use of *Cannabis* is gaining greater social acceptance for its beneficial medicinal and recreational uses. With this acceptance has come new opportunities for crop management, selective breeding, and the potential for targeted genetic manipulation. However, as an agricultural product *Cannabis* lags far behind other domesticated plants in knowledge of the genes and genetic variation that influence plant traits of interest such as growth form and chemical composition. Despite this lack of information, there are substantial publicly available resources that document phenotypic traits believed to be associated with particular *Cannabis* varieties. Such databases could be a valuable resource for developing a greater understanding of genes underlying phenotypic variation if combined with appropriate genetic information. To test this potential, we collated phenotypic data from information available through multiple online databases. We then produced a *Cannabis* SNP database from 845 strains to examine genome wide associations in conjunction with our assembled phenotypic traits. Our goal was not to locate *Cannabis*-specific genetic variation that correlates with phenotypic variation as such, but rather to examine the potential utility of these databases more broadly for future, explicit genome wide association studies (GWAS), either in stand-alone analyses or to complement other types of data. For this reason, we examined a very broad array of phenotypic traits. In total, we performed 201 distinct association tests using web-derived phenotype data appended to 290 uniquely named *Cannabis* strains. Our results indicated that chemical phenotypes, such as tetrahydrocannabinol (THC) and cannabidiol (CBD) content, may have sufficiently high-quality information available through web-based sources to allow for genetic association inferences. In many cases, variation in chemical traits correlated with genetic variation in or near biologically reasonable candidate genes, including several not previously implicated in *Cannabis* chemical variation. As with chemical phenotypes, we found that publicly available data on growth traits such as height, area of growth, and floral yield may be precise enough for use in future association studies. In contrast, phenotypic information for subjective traits such as taste, physiological affect, neurological affect, and medicinal use appeared less reliable. These results are consistent with the high degree of subjectivity for such trait data found on internet databases, and suggest that future work on these important but less easily quantifiable characteristics of *Cannabis* may require dedicated, controlled phenotyping.

## Introduction

One of the major goals of plant breeding research in domesticated species is to understand the genetic basis of traits important in their biology or commercial utilization [1, 2]. This knowledge can lead to strategies for improving yields or nutritional quality, reducing susceptibly to herbivores and pathogens, or facilitating easier growing and harvest. Of particular utility are association studies whereby segregating genetic variation among strains or individuals of a particular crop are found to correlate with one or more phenotypes of interest [3, 4]. Knowledge of such genetic variation can improve our understanding of underlying molecular pathways that influence phenotypic traits, and can be important targets for genetic manipulation.

Plant breeders have focused on a large diversity of domesticated plant species and there are hundreds of studies using association approaches to understand the genetic or genomic basis of crop development and phenotypic traits [5, 6]. One benefit of examining the underlying genetic basis of phenotypic variation in domesticated plants is that such variation can be designated clearly and precisely under carefully controlled conditions, making these plants particularly amenable to genome wide association studies (GWAS, [4, 6]). Domesticated plants also have a long and rich history of phenotypic observation producing a strong foundation on which contemporary GWAS can be based [5].

Starting in the 1970s and 80s with national laws to decriminalize possession of small amounts of *Cannabis* (aka ‘marijuana’), there has been rapid acceptance of this plant for medicinal and recreational purposes. Accordingly, it has transitioned from an ‘illegal weed’ to a valuable agricultural product, with predicted retail sales for 2021 estimated to be worth $11.9 to $17.1 billion dollars in the United States alone [7]. Despite this shift in acceptance, as well as clear monetary incentives, *Cannabis* lags behind other agricultural products in our understanding of its underlying genetic architecture and how genetic variation influences phenotypic variation [8]. A large reason for this lag is attributable to a lack of clear and consistent knowledge of phenotypes within and between *Cannabis* strains [9].

There are hundreds of distinctly named *Cannabis* strains in existence. However, given its history of elicit use, it remains unclear how much overlap among these strains exists [10]. Furthermore, many of these ‘strains’ are likely to be early generation hybrids between older varieties of *Cannabis* [11]. Regardless of the number of true-breeding, distinct strains of *Cannabis* in cultivation today, it is clear that among these plants there exists a good deal of phenotypic variation [12]. Of most interest is variation in the relative proportions of tetrahydrocannabinol (THC) and cannabidiol (CBD), as these chemicals are what give this plant most of its medicinal and psychotropic qualities. Understanding variation in plant production of other compounds such as cannabinol (CBN) is also of some importance [13].

The likelihood of finding genetic variation that correlates with a phenotype of interest depends on a number of factors, but one of the most important is the quality of the phenotypic data. More specifically, it is essential that described phenotypes of genotyped samples are accurately characterized, if confident genetic associations are to be found [14]. To date, there have been few efforts to systematically phenotype different strains of *Cannabis*. What little work has been done have focused on chemical compositions, particularly THC, CBD, and CBN quantity [15–17]. However, there is substantial trait information for *Cannabis* strains available online through various commercial and open websites (some of which are listed in the Materials and Methods section). The information on these websites ranges from THC, CBD, and CBN quantities to the impact of the strain on individual mental state to taste or odor of the strain. This information is available for a wide number of individual strains and can be collated by viewing the websites. It is possible that *Cannabis* may even have some benefit over traditional crops regarding phenotypic data availability due to the greater spirit of openness and historical sharing of information among *Cannabis* growers [18].

In this paper we examine the utility of publicly available phenotypic information provided by growers and breeders for understanding the genetic architecture of traits important in *Cannabis* agricultural production and use. It was not our goal to explicitly perform genetic association studies to locate genetic variation that correlates with specific phenotypic traits. Rather our aim was to employ a GWAS analytical framework to test the feasibility of using available online phenotypic data in future work, possibly in conjunction with other datasets. Our ability to locate statistically robust genetic variants associated with one or more phenotypes was considered as evidence in support of this aim. If and when we did locate significant associations with a phenotype, we secondarily wanted to assess the ‘reasonability’ of these associations. Such reasonability was determined based on physically proximate genes (to the variant of interest) and a review of these genes’ known biological function(s) in *Cannabis* or other plant species. Finally, we wanted to compare different categories of phenotypic data to determine if some traits are more amenable to GWAS than others, when considering the use of publicly available strain phenotypic information.

To these ends, we produced 201 web-based phenotypic character catalogs, 46 of which were based on quantitative measures of chemical content, specifically THC, CBD and CBN. The other 155 trait catalogs included 29 growth phenotypes (e.g. height, grow area, yield, grow time, etc.), 80 physiological or neurological affect phenotypes (e.g. paranoia, dry mouth, etc.), 18 taste phenotypes (e.g. ‘orange’, ‘lemon’, ‘berry’, etc.), eight phenotypes tied to origin of strain (sativa vs. indica), and 30 medicinal uses (e.g. headaches, cramps, insomnia, etc.). We then used publicly available next-generation sequence data from 845 distinct *Cannabis* strains to produce a single nucleotide polymorphism (SNP) database for testing genetic associations in conjunction with our 201 scored phenotype catalogs.

## Materials and methods

### Generation of the phenotypic trait data set

We manually collected phenotypic traits from three popular *Cannabis* websites: ‘I Love Growing Marijuana’ (ILGM; https://www.ilovegrowingmarijuana.com/), CannaSOS (CSOS; https://cannasos.com/) and Leafly (Lly; https://www.leafly.com/). We also examined the websites Grow Marijuana (https://grow-marijuana.com/), NCSM (https://www.ncsm.nl/english/), Allbud (https://www.allbud.com/), Wikileaf (https://www.wikileaf.com/) and Cannabis.info (https://www.cannabis.info/en/), for information on *Cannabis* strain phenotypes, but settled on the three sites mentioned above because of maximum overlap of strains and complete phenotypic datasets. All of the websites had substantial amounts of data for THC, CBD and CBN content in different strains as well as anecdotal information about medicinal uses, adverse effects and taste. There was also information on the growing conditions and growth traits of many strains. No two websites completely overlapped in the data they provided, and THC, CBD, and CBN levels for a given strain often varied from website to website. Additionally, there was some variability for the data on growth traits as well as anecdotal information.

We examined the three above mentioned websites for information in several trait categories for as many strains as possible that overlapped with strains for which we had genetic data (see below). Next, we scored and compiled strain-specific phenotypes for these traits (traits and our scoring system phenotypes are given in S1 Table). In all, of the 845 *Cannabis* strains for which we had genetic data, there were 290 strains that also had consistent and extensive phenotype data (S2 Table).

For THC, CBD and CBN content we performed two kinds of analyses. First, we analyzed the given values for CBD, THC, CBN and the ratio of CBN:THC (called ‘ratio’ in our tables) for each website individually. Second, where possible, we averaged values across websites (i.e., either three of three, or two or three websites, depending on strain information available). We examined associations for non-chemical traits from each website individually.

### Generation of SNP database

Paired reads from targeted amplicon sequencing of genomic DNA from 845 *Cannabis* samples have been made available by Phylos Bioscience (https://phylos.bio/), under BioProject PRJNA347566 (NCBI-SRA; https://www.ncbi.nlm.nih.gov/sra). These samples were all sequenced on an Illumina NextSeq 500. We downloaded the raw reads for each sample using the ‘fastq-dump’ function of the SRA Toolkit (v. 2.8.1–3). After downloading, we used Trim Galore (https://github.com/FelixKrueger/TrimGalore) to remove sequencing adaptors and trim bases from read ends with quality scores (Q score) less than 20. Next, we removed reads that were less than 30 bases long, in addition to any resulting unpaired reads.

To produce our variant dataset for association analyses, we first mapped the trimmed reads to the *Cannabis sativa* reference genome ASM341772v2 (genome accession number: GCA_003417725.2, [19]) using the program BWA-MEM v. 0.7.15 with default settings [20]. We then identified and removed read duplicates using the tool MarkDuplicates from Picard v. 1.77 (http://broadinstitute.github.io/picard/). This was followed by indel realignment using IndelRealigner from the Genome Analysis Toolkit (‘GATK’) v. 3.8 [21]. Independently for each sample, we called variant sites using GATK’s HaplotypeCaller (specific flags:—emitRefConfidence GVCF,—variant_index_type LINEAR,—variant_index_parameter 128000 -rf BadCigar). The resulting gVCFs (one per sample) were then combined and the samples collectively genotyped using GATK’s GenotypeGVCFs function. This gave us 84,503 raw variants in total.

After the samples were combined, we removed any variant that was not a bi-allelic, single nucleotide polymorphism (SNP). This left us with 61,393 variants. We then removed SNPs that had a quality by depth less than 20 (QD < 20.0), Fisher strand bias greater than 60 (FS > 60.0), mapping quality less than 45 (MQ < 45.0), mapping quality rank sum less than -0.2 (MQRankSum < -0.2), read position rank sum less than -1 (ReadPosRankSum < -1.0), and a strand odds ratio greater than 2 (SOR > 2.0). All filtering options were based on the developer’s recommended cutoffs, with more stringent adjustments for FS, MQ, MQRankSum and ReadPosRankSum based on the observed distributions for these parameters. Finally, we used PLINK v. 1.90b6.6 [22] to remove linked SNPs with a pairwise squared correlation (r^2^) greater than 50% within sliding windows of 50 SNPs at 10 SNP increments between windows [23]. The remaining dataset of 4,351 SNPs was used for all subsequent association analyses.

### Association analyses

Our phenotype trait matrix was combined with our SNP data for analysis with Plink v. 1.90b6.6 [22]. We then performed basic association tests comparing allele frequencies in a ‘case’ and ‘control’ framework. To establish what constituted the case vs. control for each phenotype, we used a reciprocal approach whereby we sequentially assigned the top 5%, 10%, 25% and 50% of a phenotype as the case samples and all other samples as the controls. We also performed the reciprocal analyses, sequentially examining the bottom 5%, 10%, 25% and 50% of the phenotype for the same characterizations. A list of the Plink runs we performed can be found in the S3 Table.

### Analysis of association results

For this study, we considered two major factors that could contribute to the false detection of associations, or conversely failure to detect an association. First, the phenotype as presented might be too complex or ambiguous for consistent results across strains (for example, “paranoid”, S1 Table). This would likely result in a failure to detect any associations with our generated SNP dataset. Second, the SNP data itself might not be of sufficiently high quality, failing to include the genetic variation needed for associations to be made. As the original genetic data derives from targeted amplicon sequencing, it is almost certain that important genetic variation contributing to phenotypic differences between strains is absent from our analyses. While there was little we could do to resolve this second issue, we did attempt to resolve the first concern. Specifically, as phenotypic data for the strains we used here was somewhat sparse (as indicated by the case sizes), we applied four different approaches to the discovery of significant associations (henceforth, referred to as ‘Methods 1–4’).

#### Basic association analyses (Method 1)

Although it is controversial, the statistical cutoff for significance of a GWAS is traditionally placed at p ≤ 5x10^-8^ [24–26]. However, in this study we wanted to compare the association of SNPs with phenotypes having some quantitative precision (e.g. CBD and THC content) to potentially imprecisely defined phenotypes (e.g. effects, tastes, etc.). As our primary goal was to assess whether associations were possible to make with the datasets used here, we initially set our association cutoff value to a more conservative value of p ≤ 10^−9^, recognizing that this would likely reduce our ability to identify variants of smaller effect [24]. This cutoff value attempts to balance the retention of true positives while eliminating false positives and is a good threshold for association discovery in many cases. These basic analyses constitute our ‘Method 1’ for discovering significant associations.

#### Significance within phenotype categories (Methods 2 & 3)

Another potential indication of a true, biological association is the frequency that a particular SNP is identified as significant in separate tests. Separate tests in this study would be tests involving information from different websites, or tests involving different coding schemes. For example, a SNP identified in one test for CBD is likely to be less biologically relevant than a SNP identified in multiple tests involving CBD. There are two ways to determine such overlap. The first is within specific tests for a particular trait category and the second is more broadly across categories. In the first case, all SNPs with significant scores at or below the cutoff (p ≤ 10^−9^) appearing in three or more tests for a particular phenotypic subcategory (e.g., THC content, taste, etc.) were considered significant (‘Method 2’). Subcategories are listed in Table 1. In the second case, all SNPs that were significant three or more times at the cutoff (p ≤ 10^−9^) for any test associated with a phenotype either within the chemical or non-chemical categories were considered (‘Method 3’). A comparison of SNPs found to be significant via Method 3 also allowed us to consider SNPs with potential pleiotropic effects (i.e. these genetic variants could potentially be influencing more than one phenotype). To do this, for each SNP found to be significant via Method 3, we determined whether it appeared in more than one phenotypic test subcategory (as indicated in Table 1).

**Table 1:** Phenotype categories.

Category	Number of tests
**Major category 1 –Chemical**	**49**
Subcategory 1.1 –CBD content	14
Subcategory 1.2 –THC content	11
Subcategory 1.3 –CBN content	7
Subcategory 1.4 –ratio of CBD to THC	17
**Major category 2 –non-Chemical**	**165**
Subcategory 2.1 –Growth traits	29
Subcategory 2.2 –Taste traits	18
Subcategory 2.3 –Affect traits	80
Subcategory 2.4 –Medical traits	30
Subcategory 2.5 –Sativa/Indica traits	8

Chemical and non-chemical phenotypic categories examined in this study for association methods 2 and 3. Also given are the number of tests run for each (Method 2). For the two major categories (chemical and non-chemical; Method 3), the number of tests run is the sum of each subcategory for that phenotype.

#### Subsampling and bootstrap analyses (Method 4)

It is well established that imbalances in the numbers of case and control samples may affect association study results [27, 28]. To partially account for this, we used a bootstrap approach to generate subsampled control datasets with equal numbers of case and control samples. Samples were randomly selected without replacement using a custom Perl script, utilizing a Fisher Yates shuffle [29]. We then ran an association test comparing allele frequencies between the case and control samples in Plink. This subsampling and subsequent association analysis was run 100 times and variants found to have significant associations with a phenotype (with a threshold of p ≤ 10^−9^), in at least 95% of the runs were considered further (‘Method 4’).

### Biology of significant SNPs

#### Genes in proximity to significant SNPs

For any SNPs with a phenotypic association that met or exceeded our significance threshold, we next used information from the annotated *Cannabis* reference genome (*Cannabis sativa*, ASM341772v2; accession number: GCA_003417725.2) to locate all genes within 25 Kb both upstream and downstream of the variant (50 Kb windows in total). In some cases, there were no annotated genes within this span, while in other cases there were as many as seven genes. When one or more genes were present, we conducted a literature survey to assess the likelihood that the gene(s) could have an effect on our phenotype of interest.

#### Chromosomal location of significant SNPs

The causative gene or genes for a specific trait can be as distant as two million base pairs from the associated SNP [30]. Therefore, we also wanted to examine which chromosomes that significantly associated SNPs were found on. In particular we were interested in the known chromosomal locations of genes involved in THC and CBD synthesis in *Cannabis* [31, 32]. For all significantly associated SNPs we determined its level of significance and the chromosome it was located on. For chemical traits we clustered SNPs into those related to THC content, CBD content, CBN content, and the ratio of THC:CBD. For non-chemical traits we clustered the SNPs into ‘side effect’ phenotypes, ‘taste’ phenotypes, ‘medical’ phenotypes, ‘growth’ phenotypes, and *sativa*:*indica* hybrid ratio.

#### GO term analysis

To further examine how phenotypes may be impacted by the genetic variation identified, we utilized all genes found to be within 25Kb of a significantly associated SNP (Method 1). This gene list was compiled using UNIPROT (https://www.uniprot.org/), and then fed into the Panther gene list analyzer (http://www.pantherdb.org/). We examined the gene categories and GO annotations for the categories using Panther by generating histograms of the nearest child terms (i.e. more narrowly defined categories) in four parent GO categories: ‘molecular function’, ‘cellular component’, ‘biological process’, and ‘protein class’. We also examined GO terms that were over-represented to determine if any categories of genes showed strong genetic associations with phenotypes. This was done in Panther, with Fisher’s exact test and Bonferonni correction.

## Results and discussion

The genomics of *Cannabis* is becoming an increasingly active research topic in plant biology [9, 33–41]. These studies have used genomic level information (e.g. whole genome sequencing, targeted genome sequencing, transcriptome sequencing, etc.) to explore the role of genetic variation in differences observed among the hundreds of distinct *Cannabis* strains. This research has predominately relied on correlated phenotyping of the sequenced plants, a reasonable but challenging task for large numbers of different strains. Furthermore, the traits investigated have predominately been readily quantifiable phenotypes such as chemical composition. Less well studied are more qualitative phenotypes such as taste and mental state alteration. Such phenotypic traits likely present a greater challenge for determining correlative genetic variation associated with them. However, given the uses of *Cannabis* for medicinal and recreation purposes, variation of such traits will remain important to understand at a genetic level.

The goals of this study were threefold. First, we wanted to establish if online databases providing *Cannabis* phenotypic data are sufficiently consistent to allow significant genetic associations to be found. Secondly, we wanted to assess whether any candidate SNPs uncovered were ‘reasonable’ as determined by their genomic location in proximity to genes known to influence the correlated phenotype in *Cannabis* or other plant species. Predominately, we expected that a phenotypic data set with little or no utility would show either a lack of association at any level or associations that, based on genomic position and gene proximity, had little or no support in the literature for a probable influence on the phenotype of interest. Conversely, high-quality phenotypic data should produce confident associations in genomic regions with genes likely to influence the focal phenotype. Finally, we wanted to determine what types of phenotype traits (e.g. chemical vs. non-chemical), were most amenable to performing association studies in a genome-wide context with information from available online databases.

### Factors effecting the utility of internet phenotypes

**Fig 1:**
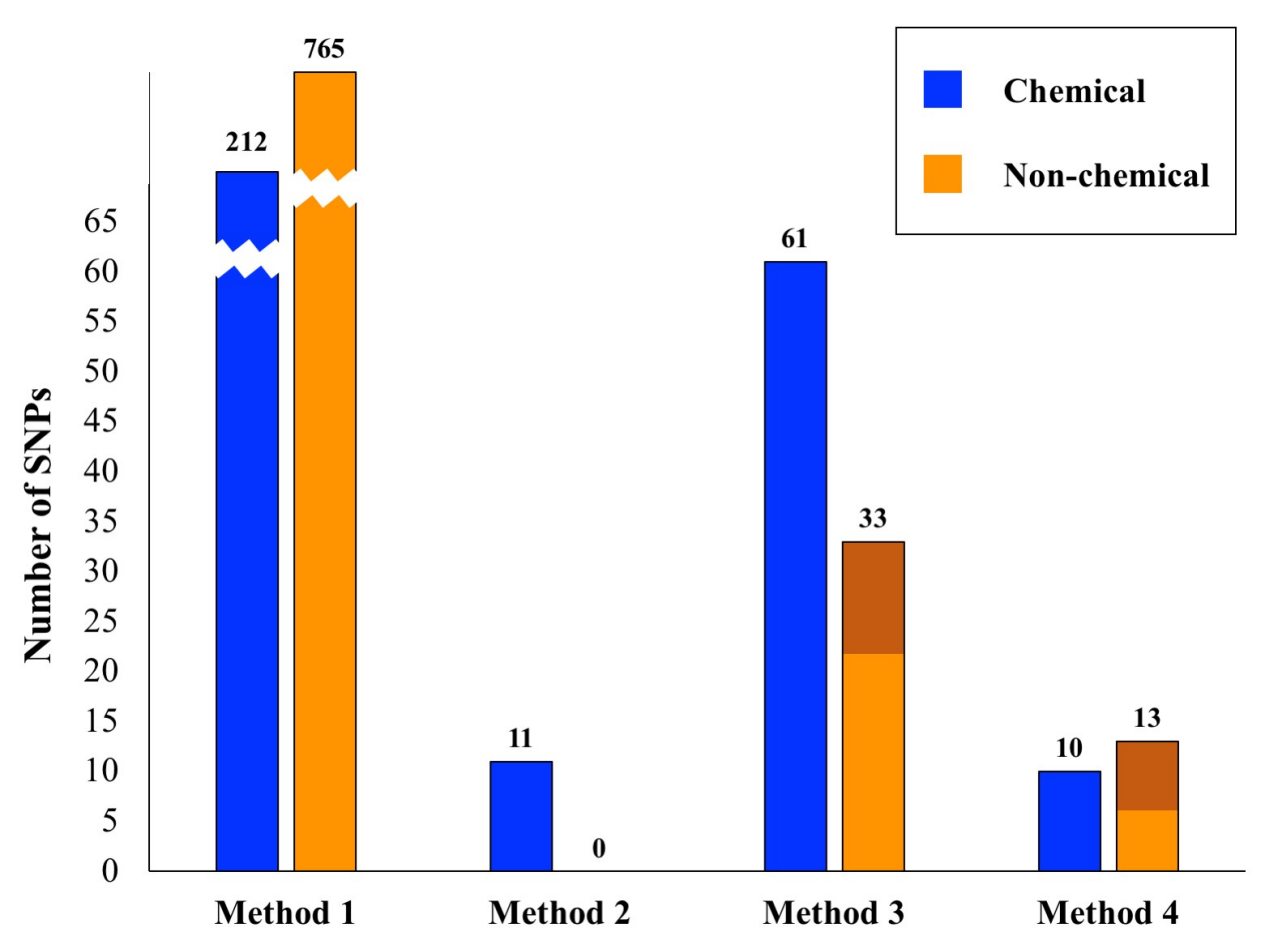
Significant SNPS relative to analytical method. Shown are the numbers of significant SNP associations for the four methods outlined in the text. For Method 1 the bars are broken for convenience of display. The number of significant SNPs for each Method/category combination is given above its respective bar. The dark orange shading for Methods 3 and 4 in the non-chemical category indicates the number of SNPs significant for the subcategory ‘growth traits’.

Fig 1 shows the total number of significant SNPs across all tests for each of the four association detection methods, considering chemical and non-chemical traits separately. A comparison of the results from chemical and non-chemical traits across these different methods is illustrative of the quality of internet-based phenotypic data. In our most basic approach (Method 1) we carried out simple genome-wide associations attempting to correlate *Cannabis* phenotypic variation with genetic variation in a ‘case’ vs. ‘control’ framework. We performed 201 distinct trait association tests, 155 of which were for non-chemical traits (i.e. not THC, CBN or CBD based) and 46 of which were for chemical traits (THC, CBD and CBN content; S1 & S3 Tables). For the least stringent method of extracting associations (Method 1), there were over 900 significant SNPs (p ≤ 10^−9^) across all tests, with about half of the tests yielding significant SNP associations. The number of SNPs associated with chemical and non-chemical traits was roughly proportional to the number of tests performed suggesting randomness in these results. Furthermore, within the non-chemical traits we could subdivide these into growth, affect, medicinal, and taste traits. The number of significant associations in each of these subcategories of non-chemical traits was again proportional to the number of tests within this category, further suggesting an element of randomness.

In many of our Method 1 analyses, either the case or control sample sizes were relatively small. Comparisons between the number of significant associations and sample sizes (S1 & S2 Figs) showed a U shape where phenotypes with very small case or control numbers (< 10) had a high number of SNPs correlated with the trait of interest. From our literature survey of genes around significant SNPs when the case or control samples were small, it became apparent that these variants were difficult to interpret and reasoned that associations with small case/control sizes were in many respects random. This suggests that phenotypic information with this structure has limited utility for genetic association studies.

Assessing the presence of significantly associated SNPs across multiple tests within a subcategory (Method 2), allowed us to reduce our list of ~900 SNPs considerably (Tables 2 & 3). For chemical-based tests there were 11 associated SNPs across all subcategories and none for non-chemical tests. For Method 3, when compared across tests there were 61 significantly associated SNPs for the chemical category and 33 for the non-chemical category (Tables 2 & 3). Because there were three times as many non-chemical traits as chemical traits, these results suggest that the chemical traits may be more stable for association analyses than the more anecdotal non-chemical traits. Additionally, of the 33 SNPs identified for the non-chemical traits, a third of these are linked to growth, suggesting that within the non-chemical category, plant growth data from the internet could have some utility in association studies. Considering the 94 variants found using Method 3, 29 were determined to be significant in more than one phenotypic test subcategory, suggesting possible pleiotropic effects (S5 Table).

**Table 2:** Genes with significant associations for chemical traits.

Method 2	Method 3
Uncharacterized (6X)	Uncharacterized (9X)
receptor-like protein EIX2	receptor-like protein EIX2 (2X)
protodermal factor 1-like	protodermal factor 1-like
protein FAR1-RELATED SEQUENCE	protein FAR1-RELATED SEQUENCE (2X)
FAD/NAD(P)-binding oxidoreductase family protein	FAD/NAD(P)-binding oxidoreductase family protein
CDPK-related kinase 4	CDPK-related kinase 4
	ARF guanine-nucleotide exchange factor GNL2 (2X)
	Aspartic peptidase domain containing protein
	AT-hook motif nuclear-localized protein 1-like
	Bulb-type lectin domain containing protein
	calcium-dependent protein kinase 13 (2X)
	Endochitinase-like
	Endonuclease/exonuclease/phosphatase (5X)
	Fatty Acid-binding Proteins (FABPs)
	fibroin heavy chain
	Glycoside hydrolase
	hypothetical protein (7X)
	LRR domain containing protein
	N-terminal acetyltransferase A, auxiliary subunit
	O-fucosyltransferase 28-like
	proteasome adapter and scaffold protein ECM29 (2X)
	protein NYNRIN-like (2X)
	receptor-like protein Cf-9
	Ribonuclease H-like domain containing protein
	Serine/threonine protein kinase (3X)
	Sodium/sulfate symporter
	stemmadenine O-acetyltransferase-like (2X)
	Voltage dependent potassium channel

Best gene hits in proximity to significant SNPs for chemical traits using Methods 2 and 3. All best hits were to the *Cannabis sativa* genome and annotation. The numbers in parentheses after a gene name indicate the number of significantly associated SNPs that fell near this gene (when greater than 1). Additional information for each gene is available in S4 Table.

**Table 3:** Genes with significant associations for non-chemical traits.

Method 2	Method 3
No Significant SNPs	ARF guanine-nucleotide exchange factor GNL2 (8X)
	Cysteine-rich RLK (RECEPTOR-like protein kinase) 8
	floral homeotic protein APETALA 2-like isoform X1
	myosin tail region-interacting protein MTI1-like (2X)
	neurofilament medium polypeptide
	noncoding (2X)
	nucleolar and coiled-body phosphoprotein 1-like
	probable methyltransferase PMT24 (2X)
	receptor-like protein EIX2 (4X)
	stemmadenine O-acetyltransferase-like
	tRNA (cytosine(34)-C(5))-methyltransferase-like (2X)
	tyrosine N-monooxygenase-like (2X)
	uncharacterized protein (6X)

Best gene hits in proximity to significant SNPs for non-chemical traits using Methods 2 and 3. All best hits were to the *Cannabis sativa* genome and annotation. The numbers in parentheses after a gene name indicate the number of significantly associated SNPs that fell near this gene (when greater than 1). Additional information for each gene is available in S4 Table.

When we required that a variant be significant in more than one test (Methods 2 and 3), the number of phenotype-associated SNPs found diminished substantially (Fig 1). We also saw differences comparing the consolidation of phenotypes into refined subcategories (Method 2) versus the coarser merging of phenotypes into two major categories (chemical traits and non-chemical traits; Method 3). Generally, these results indicate that broader classes of phenotypes may better facilitate the identification of biologically relevant genetic variation in *Cannabis*. However, this comes at the cost of losing precision in understanding potential molecular relationships between genotypes and phenotypes.

Our second, complementary approach to reducing the large number of significantly correlated SNPs was to repeatedly subsample the overrepresented category and normalize sample sizes between ‘cases’ and ‘controls’ (Method 4). Combining this with a bootstrapping approach allowed us to focus on another conservatively determined subset of SNPs (Fig 1, Table 4). After these bootstrap analyses, there were ten significant chemical and 13 significant non-chemical SNPs. Within the significant non-chemical SNPs, 50% were for growth traits. Again, given that there are three times as many non-chemical as chemical traits, these results indicate that chemical traits from the internet may be of sufficient quality for use in association studies. Similarly, the large proportion of significant SNPs for non-chemical growth traits indicates that this phenotype data may also be stable, and potentially useful in association studies. Given that such traits are to a large extent quantitative, this result is not surprising. Internet based phenotypic data for other non-chemical traits such as mental affect, medicinal utility and taste categories appeared inadequate for association analyses. To what extent this is due to the quality of online data versus the likely complex genetic underpinnings of such traits remains to be determined.

**Table 4:** Significant associations using Method 4.

Chemical	non-Chemical
non-coding (2X)	noncoding (2X)
receptor-like protein Cf-9 [*Cannabis sativa*]	floral homeotic protein APETALA 2-like isoform X1 [*Cannabis sativa*]
calcium-dependent protein kinase CDPK 13 (2X) [*Cannabis sativa*]	aspartate carbamoyltransferase [*Trema orientale*]
enolase-like [*Cannabis sativa*]	ornithine carbamoyltransferase
putative Peroxidase 48 [*Cannabis sativa*]	glycosyltransferase At5g20260 isoform X2 [*Cannabis sativa*]
uncharacterized protein (2X) [*Cannabis sativa*]	swi5-dependent recombination DNA repair protein 1 homolog [*Cannabis sativa*]
voltage dependent potassium channel [*Trema orientale*]^a^	midasin-like [*Cannabis sativa*]
	major facilitator [*Parasponia andersonii*]
	NEDD8-conjugating enzyme Ubc12 isoform X2 [*Cannabis sativa*]
	NYNRIN-like [*Brassica rapa*]
	gag-pol polyprotein [*Glycine max*]
	Transposon Ty3-G Gag-Pol polyprotein [*Vitis vinifera*]

List of genes from chemical and non-chemical SNPs that had significant p values and passed bootstrap tests. All chemical-associated genes were either with THC and/or CBD except ‘voltage dependent potassium channel’. The numbers in parentheses after a gene name indicate the number of significantly associated SNPs that fell near this gene (when greater than 1). In brackets [] are the species’ genome models used to identify the protein.

^a^ Association with CBN.

### SNPs associated with chemical content phenotypes

Genes we determined were associated with chemical phenotypes are given in Tables 2 and 4 (also see S4 Table). If the basic analytic approach we have used here has any biological validity, then we have established associations of THC/CBD phenotypes with several novel candidate genes annotated in the *Cannabis* genome. Four of the significant SNPs detected by Method 4 are in genes that have roles in plant physiology peripherally or are directly connected to plant secondary compound production (Table 4). The enzyme Enolase has an upstream function in THC and CBD synthesis [42]. Likewise, Peroxidase has also been implicated in the synthesis pathways of these chemicals [43]. CDPK’s and receptor-like protein Cf-9 have been implicated in plant stress systems and in this way may be implicated in plant secondary compound synthesis. All of the genes in the list were detected in tests for CBD and THC with one exception. The potassium channel gene in the list was detected using data from CBN content. While the other genes in the list are reasonable candidates for biosynthetic pathways to CBD and THC, the potassium channel gene is odd. It is interesting though that the majority of associations are detected with tests for THC and CBD and not for CBN as the latter is not a product of gene interactions in the *Cannabis* genome. Rather, CBN content is the result of environmental breakdown byproducts. We suggest here that the CBN tests could serve as a control for the other chemical tests as it should not show association with gene SNPs. In this respect it is also noteworthy that two of the associated SNPs with THC/CBD content were in non-coding regions, or positions in the genome that might turn out to be regulatory. Furthermore, many of the significant SNPs we examine here were in or near retrotransposons which are scattered throughout the *Cannabis* genome [31].

Of additional note, we did not detect SNPs directly associated with the gene Tetrahydrocannabinolic acid (THCA) synthase, which encodes the enzyme responsible for catalyzing the conversion of cannabigerolic acid (CBGA) to THC [44]. Equally compelling, we also did not detect SNPs associated with Cannabidiolic acid synthase, an enzyme that catalyzes the formation of cannabidiolate, a carboxylated precursor of cannabidiol (CBD, [45]). SNPs within or in proximity to these genes and several others involved in the synthesis of THC and CBD are present in our genetic data. If we assume that genetic variation influencing chemical phenotypes is segregating at relatively high frequency, and correspondingly, is likely to be represented in this database, then the absence of SNPs associated with these genes suggests it is not variation in these regions that directly effects the THC or CBD content of a plant. Rather we suggest that the genetic architecture of THC and CBD phenotypes is more complex than just changing the structures or regulation of the THC/CBD end products. This inference is supported by an analysis of the function of genes that are associated with the phenotypes used in this study (see below).

### SNPs associated with non-chemical phenotypes

As indicated above, non-chemical traits appeared less stable with respect to attempts to discover associations except possibly for growth traits such as height, time to flowering, yield and growth area. As a consequence, we will discuss here the SNP associations with growth traits (Tables 3 and 4). The association of a floral homeotic gene (APETALA 2) with growth phenotypes is interesting in that this gene is responsible for hormone regulation specifically in the floral structures of plants [46]. Such roles would include regulation of growth rates and other growth-related phenotypes. Several other significant SNP related traits like carbamoyletransferases and glycosyltransferase are involved in very basic cellular synthesis pathways and plant secondary metabolism. The involvement of these genes in plant growth is of interest too. Finally, several growth traits are associated with proteins involved in transposon regulation in plants (NYNRIN) or are genes in transposons themselves (gag-pol polyprotein and Transposon Ty3-G Gag-Pol polyprotein). The involvement of transposons and repetitive genome content in the phytochemistry of *Cannabis* has recently been established [41, 47, 48], and the detection of SNPs in these genomic regions could also be of biological significance.

### Chromosomal location of significant SNPs

**Fig 2:**
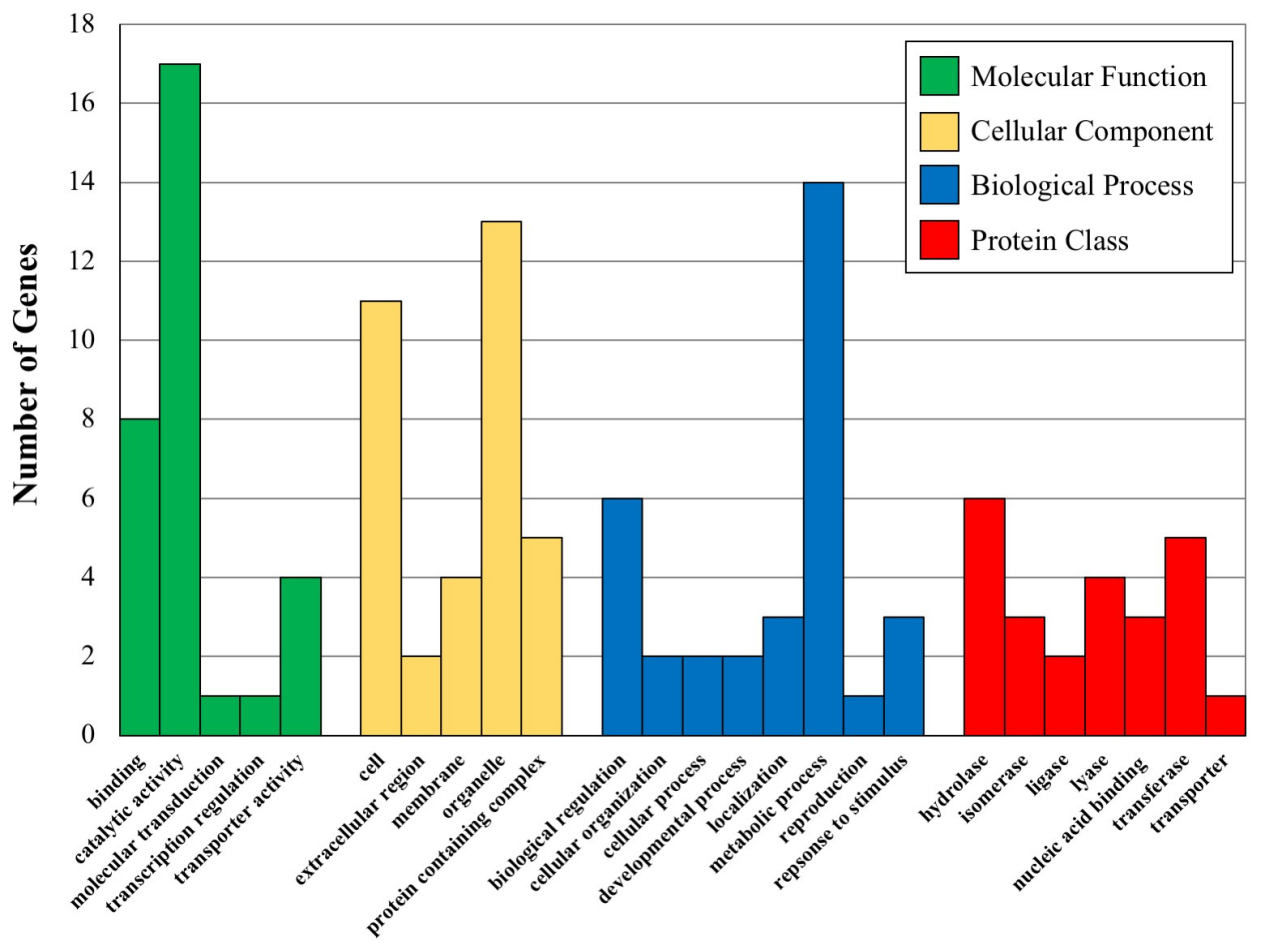
Graph of chromosomal location and SNP significance. Chromosome number is listed at the top. Below the dotted line are the top 5% most significant SNPs. The trait categories are listed in the box at the lower left along with their respective symbol colors. Chemical categories are: ‘cbd’, ‘thc’, ‘cbn’, and ‘ratio’. Non-chemical categories are: ‘I/S’ (the ratio if ‘indica’ to ‘sativa’), ‘taste’, ‘effect’, ‘medical’, and ‘grow’. At the bottom we tally the number of SNPs that have p values in the top 5% most significant SNPs (top value: chemical categories, bottom value: non-chemical categories).

Brodie and colleagues [30] demonstrated that the causative locus of a trait can be located relatively distant from the associated SNP. To examine this possibility, we analyzed the chromosomal location of all of the significant SNPs in our study. Overall, there were over 200 SNPs associated with non-chemical traits and tests and over 700 SNPs associated with chemical traits and tests. The chromosomal location of these significant traits and their significance are shown in Fig 2. When we examined the top 5% of SNPs by statistical significance, most were chemical (29 out of 40 SNPs). Interestingly, the ‘medical’ category of SNPs made up the majority of the non-chemical, highly significant SNPs (7/11). The high frequency of chemical trait associated SNPs that were highly significant is evidence that the genetic variants segregating with chemical traits were more readily detected than with non-chemical trait. We also did not detect highly significant CBN SNP associations with chromosomes, which was consistent with our earlier observations.

Three chromosomes (3, 6 and 8), harbored the majority of the chemical SNPs. This result is interesting because of the co-location of known THC and CBD synthesis genes on these chromosomes. The THCA synthase and CBDA synthase genes (genes coding for enzymes involved in the last step of THC and CBD respectively), are located on Chromosome 6 and indeed there is also considerable duplication of these genes in the *Cannabis* genome on Chromosome 6 [19, 49]. Chromosome 8 also appears to contain genes relevant to total cannabinoid content and to specific cannabinoid synthesis. Chromosome 3 is known to have genes involved in both THC and CBG synthesis. On the other hand, both Grassa et al. [31], and Welling et al. [32], suggest that Chromosome 9 could be a major chromosome affecting cannabinoid synthesis, and we find no chemical but three highly significant non-chemical trait associated SNPs on this chromosome.

### GO classification of associated SNPS

**Fig 3:**
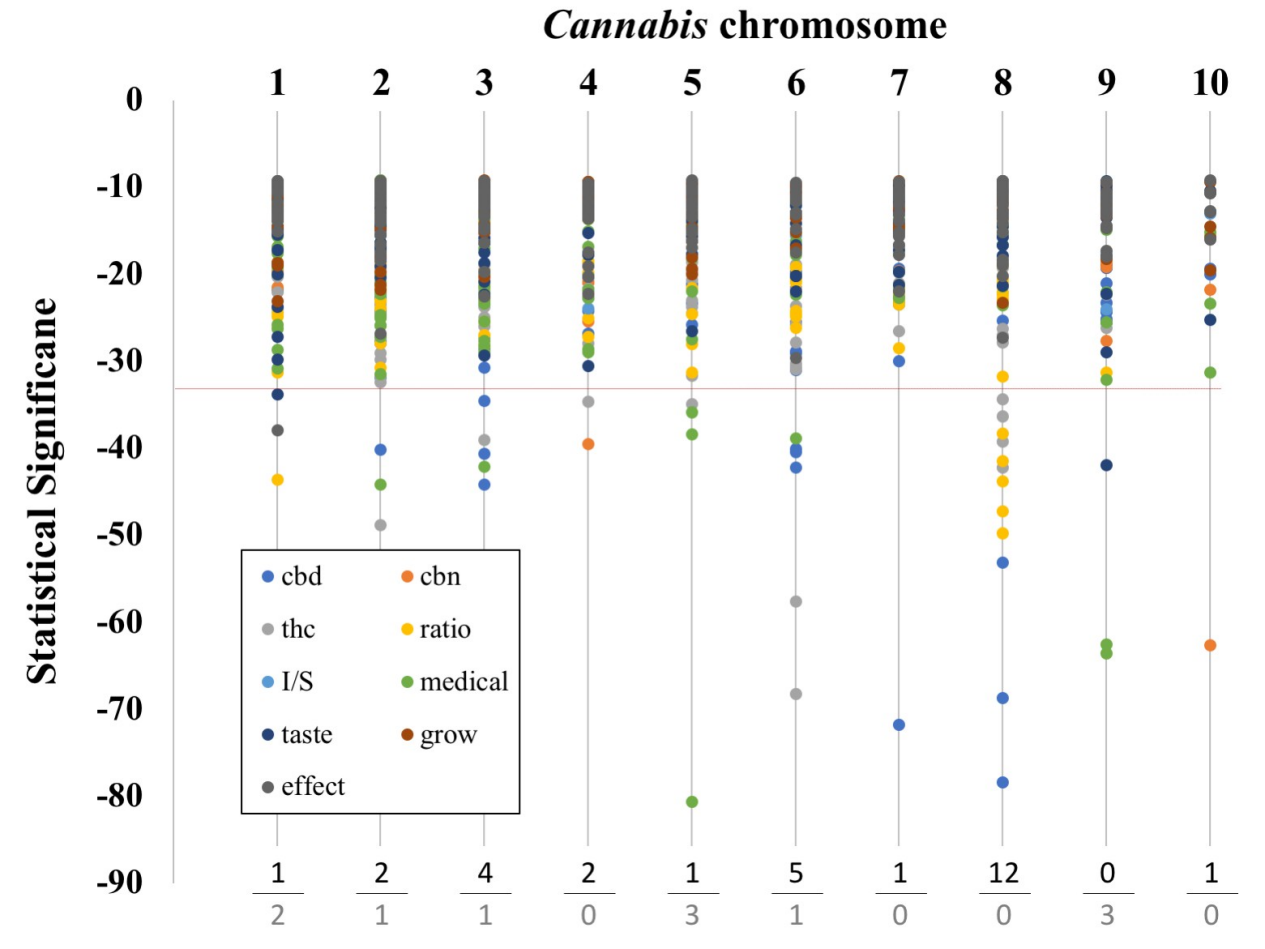
Bar graphs showing the number of proteins (Y axis) in various GO categories and protein classes for the indicated significant GO categories. Molecular Function GO terms are binding-GO:0005488, catalytic activity-GO:0003844, molecular transduction-GO:0060089-transcription regulation-GO: 0140110 and transporter activity-GO:005215. Cellular Component GO terms are Cell-GO:0005623, extracellular region-0005576, membrane-GO:0016020, organelle-GO:0043226 and protein containing complex-GO:003299. Biological Process GO terms are biological regulation-GO-0065007, cellular organization-GO0071840, cellular process-GO:0009987, developmental process-GO:0032502, localization-GO:0051179, metabolic process-GO:0008152, reproduction-GO-0000003, response to stimulus-GO:0050896. Protein Class GO terms are hydrolase-PC00121, isomerase-PC00135, ligase-PC00142, lyase-PC00144, nucleic acid binding-PC00171, transferase-PC00220 and transporter-PC00227.

Using the large number of significantly associated SNPs found using Method 1, we combined the genes associated with these SNPs and categorized the related GO terms. The distribution of children GO categories linked to these genes are shown in Fig 3, where the most abundant GO terms for the four parent categories are plotted against the number of times they appear in the gene lists. These results demonstrate interesting trends for the associated genes of both major categories of phenotypes. For instance, the top two molecular function children GO terms are ‘binding’ and ‘catalytic activity’, both of which are important in gene regulation. The top two biological process children terms are ‘metabolic process’ and ‘biological regulation’, both of which are involved in regulating the amount of gene product. Finally, ‘hydrolases’ and ‘oxidoreductases’ are the two most represented protein classes and both of these classes of proteins are involved in plant secondary compound metabolism.

We also tested the list for over-representation of GO terms in the GO parent categories. The Cellular Component parent category had no significant over-representation, while Molecular Function and Biological Process did at two-fold enrichment. The Biological Process over-representation is for ‘cellular metabolic processes’ and the Molecular Function over-representation is for ‘organellar processes’. These results suggest that regulation of chemical composition (THC and CBD) may be connected to the cellular metabolic processes of the plant cells and also associated with organellar function. It is well known that both THC and CBD have effects on and in organelles, particularly mitochondria where they bind to membranes and disrupt mitochondrial function [50]. It is possible that the suite of genes we show associations with THC and CBD levels are involved in mediating or regulating organellar function when interacting with these chemicals.

## Conclusions

This study set out to assess the potential utility of web-based information from a large number of *Cannabis* strains for determining genetic associations with some of this plant’s important phenotypic traits. We found that internet sources of strain-specific information that is readily quantifiable such as chemical composition (i.e. THC and CBD content) and growth traits might be potentially useful for association studies. However, phenotypic information that is more qualitative or relies on anecdotal descriptions is likely insufficiently precise for useful association analyses. This result suggests that more precise measurement of traits such as yield, medicinal, or side effects would be needed in order to perform genetic association studies for *Cannabis* non-chemical traits.

If the associations we have detected for chemical traits like THC and CBD content using this approach have any biological significance, then we can also conclude that genetic variation influencing these phenotypes occurs outside the genes within the synthetic pathways of THC and CBD. The genes effected by these variants may be involved in the quantity of these chemicals produced by *Cannabis* plants, or might be involved in more mundane cellular processes like organellar properties or cellular metabolism properties. At the very least, the SNPs we detected for chemical (and a handful of non-chemical traits) may be linked to the known cannabinoid synthesis genes on Chromosomes 3, 6 and 8.

Overall, web based chemical content data appears to have potential utility for gene association studies considering THC and CBD content. In the future, more directed and precise chemical concentration data might enhance detection of other chemical traits such as ratios and CBN content. Such data will additionally provide more precise inferences for the traits successfully examined here. Some such studies of chemical makeup have been reported [16] and these should add more precision to association studies in the future. As well as dedicated phenotyping for the purposes of association studies, web-based data may also continue to have utility in this field. Specifically, websites should make a concerted effort to work with growers and other interested parties to compile the best and most precise information possible.

## Supporting information

S1 Fig Graph of case size (X axis) versus -log of significance level of SNPs in this study. We have divided the space into Case Size slivers. A is between 0 and 10, B between 10 and 25, C between 25 and 50, D between 50 and 100 and E between 100 and 150.
(TIF)

S2 Fig Graph of percent of cases versus percent significant (cutoff set at p < 10−9; see text). The boxes indicate two ranges over which we are confident of reasonable results. The read box indicates the range of the most stringent approach we took.
(TIF)

S1 Table Phenotypes and scoring system. The phenotypic traits examined in this study, classified by category. Also given are the binary (case vs. control) trait scoring definitions used for each phenotype.
(XLSX)

S2 Table Cannabis strains. The list of *Cannabis* strains used for trait associations in this study. The strains listed had both phenotypic data available through one or more of the websites listed in the methods, as well as genetic data available. Also given is each strain’s NCBI-SRA BioSample ID (https://www.ncbi.nlm.nih.gov/sra).
(XLSX)

S3 Table Association test information. The websites, traits and phenotypes of the 201 basic association tests performed in this study. The sizes of the case category and the p values of the most significant SNP (‘most sign’) are also given in the table.
(XLSX)

S4 Table Significant SNP data. Extended data for all SNPs determined to be significant using Methods 2 and 3.
(XLSX)

S5 Table Possible pleiotropic SNPs. SNPs determined to be significantly associated with more than one phenotypic trait (from Method 3).
(XLSX)
