# Correction: Can public online databases serve as a source of phenotypic information for *Cannabis* genetic association studies?

**DOI:** 10.1371/journal.pone.0251930

**Published:** 2021-05-13

**Authors:** Matthew L. Aardema, Rob DeSalle

The captions for Figs 2 and 3 are incorrectly switched. The caption that appears for Fig 2 should be for Fig 3, and the caption that appears for Fig 3 should be for Fig 2. The figures appear in the correct order. Please see the complete, correct Figs 2 and 3 captions here.

**Fig 2:**
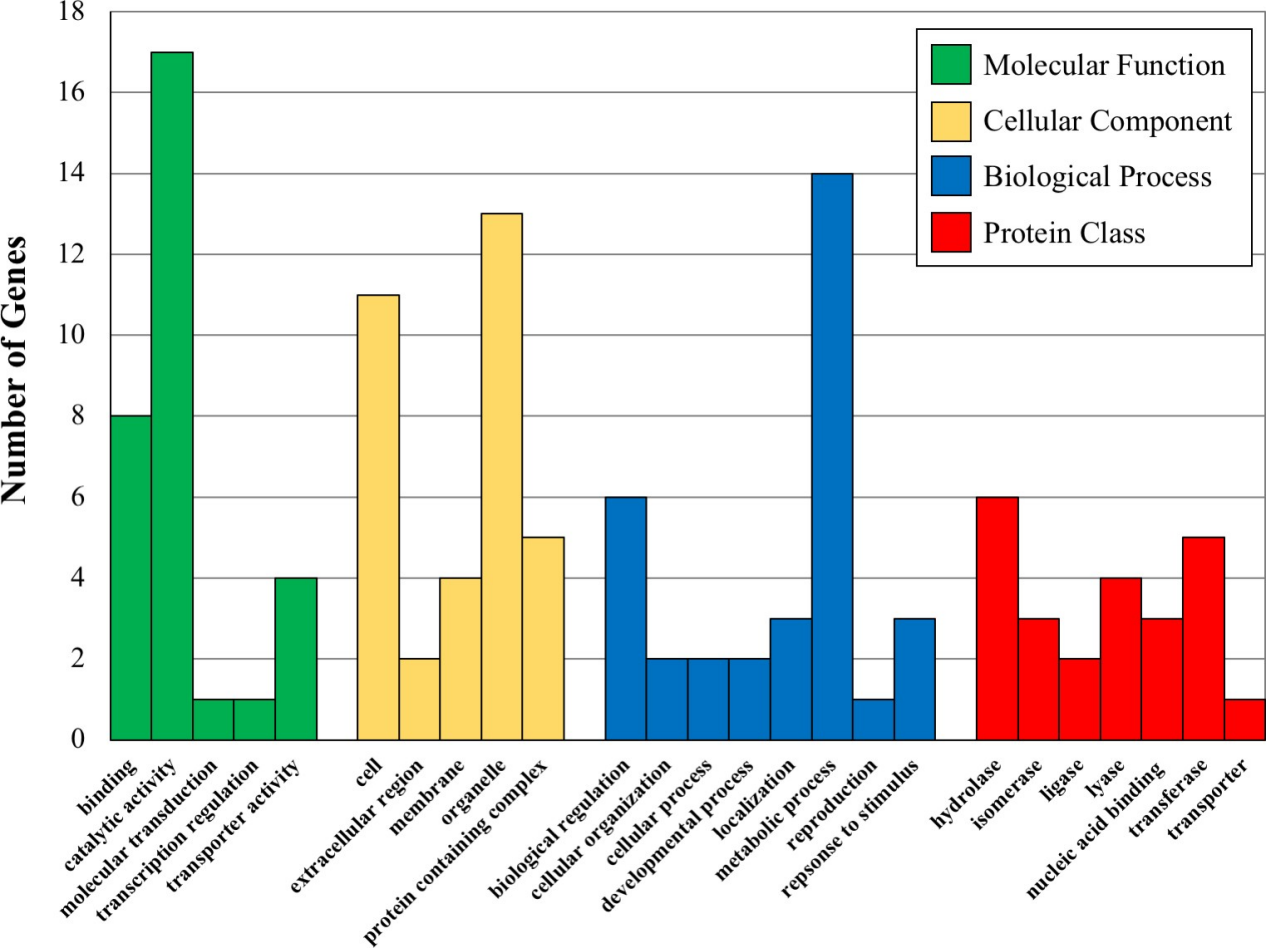
Bar graphs showing the number of proteins (Y axis) in various GO categories and protein classes for the indicated significant GO categories. Molecular Function GO terms are binding-GO:0005488, catalytic activity-GO:0003844, molecular transduction-GO:0060089-transcription regulation-GO: 0140110 and transporter activity-GO:005215. Cellular Component GO terms are Cell-GO:0005623, extracellular region-0005576, membrane-GO:0016020, organelle-GO:0043226 and protein containing complex-GO:003299. Biological Process GO terms are biological regulation-GO-0065007, cellular organization-GO0071840, cellular process-GO:0009987, developmental process-GO:0032502, localization-GO:0051179, metabolic process-GO:0008152, reproduction-GO-0000003, response to stimulus-GO:0050896. Protein Class GO terms are hydrolase-PC00121, isomerase-PC00135, ligase-PC00142, lyase-PC00144, nucleic acid binding-PC00171, transferase-PC00220 and transporter-PC00227.

**Fig 3:**
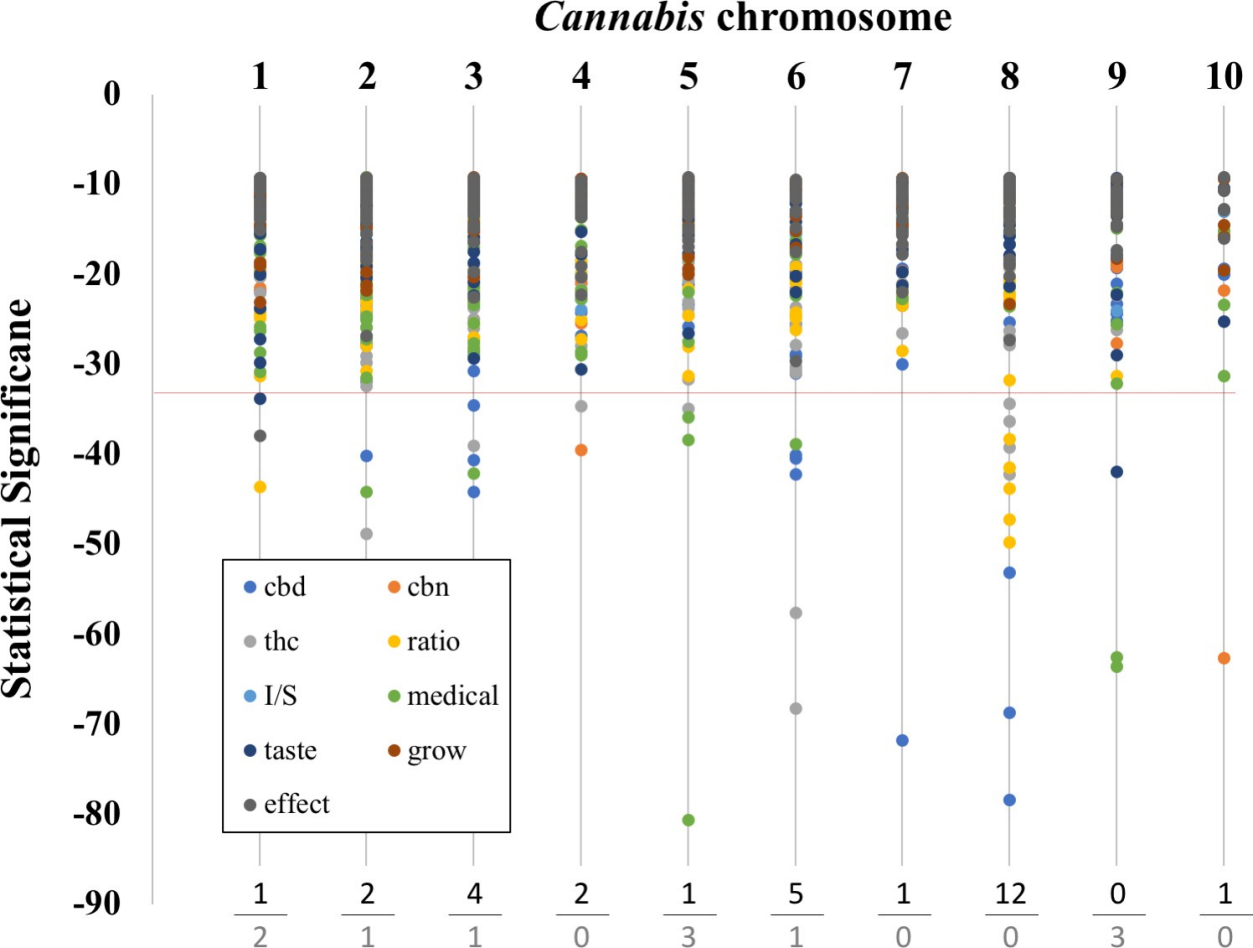
Graph of chromosomal location and SNP significance. Chromosome number is listed at the top. Below the dotted line are the top 5% most significant SNPs. The trait categories are listed in the box at the lower left along with their respective symbol colors. Chemical categories are: ‘cbd’, ‘thc’, ‘cbn’, and ‘ratio’. Non-chemical categories are: ‘I/S’ (the ratio if ‘indica’ to ‘sativa’), ‘taste’, ‘effect’, ‘medical’, and ‘grow’. At the bottom we tally the number of SNPs that have p values in the top 5% most significant SNPs (top value: chemical categories, bottom value: non-chemical categories).
