# Supplementary figures and images for: Can public online databases serve as a source of phenotypic information for Cannabis genetic association studies?
Source: PLoS One. 2021 Feb 23;16(2):e0247607. doi: 10.1371/journal.pone.0247607 (PMC7901747; doi:10.1371/journal.pone.0247607)

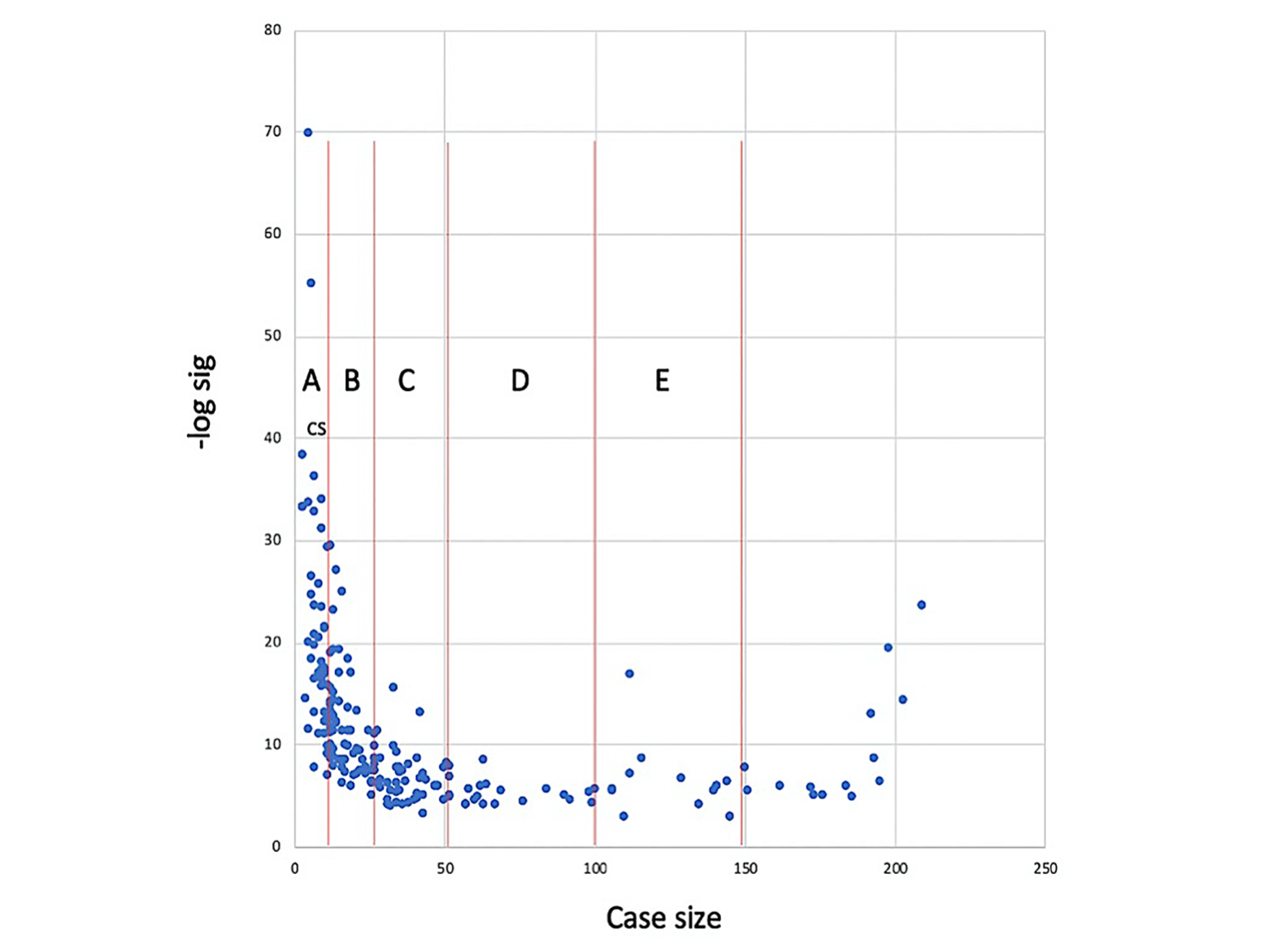

Supplement: S1 Fig — We have divided the space into Case Size slivers. A is between 0 and 10, B between 10 and 25, C between 25 and 50, D between 50 and 100 and E between 100 and 150. (TIF) [file pone.0247607.s001.tif]

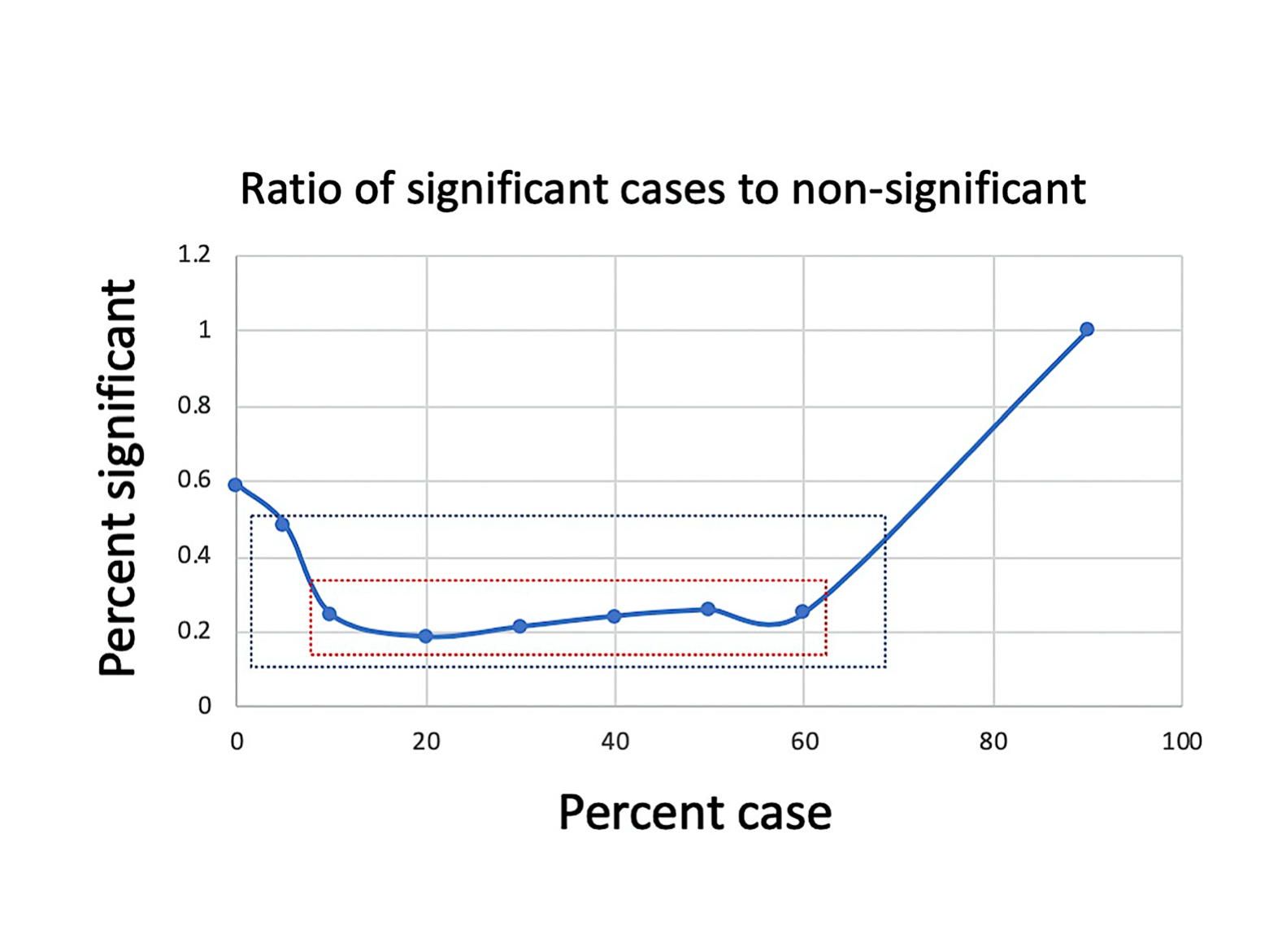

Supplement: S2 Fig — The boxes indicate two ranges over which we are confident of reasonable results. The read box indicates the range of the most stringent approach we took. (TIF) [file pone.0247607.s002.tif]
